# Functional validity, role, and implications of heavy alcohol consumption genetic loci

**DOI:** 10.1126/sciadv.aay5034

**Published:** 2020-01-15

**Authors:** Andrew Thompson, James Cook, Hélène Choquet, Eric Jorgenson, Jie Yin, Tarja Kinnunen, Jeff Barclay, Andrew P. Morris, Munir Pirmohamed

**Affiliations:** 1 Wolfson Centre for Personalised Medicine, Institute of Translational Medicine, University of Liverpool, Liverpool, UK.; 2 MRC Centre for Drug Safety Science, Institute of Translational Medicine, University of Liverpool, Liverpool, UK.; 3 Liverpool Centre for Alcohol Research University of Liverpool, Liverpool, UK.; 4 Biostatistics, Institute of Translational Medicine, University of Liverpool, Liverpool, UK.; 5 Division of Research, Kaiser Permanente Northern California, Oakland, CA 94612, USA.; 6 Department of Biological and Geographical Sciences, School of Applied Sciences, University of Huddersfield, Huddersfield, UK.; 7 Cellular and Molecular Physiology, Institute of Translational Medicine, University of Liverpool, Liverpool, UK.

## Abstract

High alcohol consumption is a risk factor for morbidity and mortality, yet few genetic loci have been robustly associated with alcohol intake. Here, we use U.K. Biobank (*n* = 125,249) and GERA (*n* = 47,967) datasets to determine genetic factors associated with extreme population-level alcohol consumption and examine the functional validity of outcomes using model organisms and in silico techniques. We identified six loci attaining genome-wide significant association with alcohol consumption after meta-analysis and meeting our criteria for replication: *ADH1B* (lead SNP: rs1229984), *KLB* (rs13130794), *BTF3P13* (rs144198753), *GCKR* (rs1260326), *SLC39A8* (rs13107325), and *DRD2* (rs11214609). A conserved role in phenotypic responses to alcohol was observed for all genetic targets available for investigation (*ADH1B, GCKR, SLC39A8*, and *KLB*) in *Caenorhabditis elegans*. Evidence of causal links to lung cancer, and shared genetic architecture with gout and hypertension was also found. These findings offer insight into genes, pathways, and relationships for disease risk associated with high alcohol consumption.

## Teaser

We identify six genetic loci associated with heavy alcohol intake in humans, and provide functional validation in *C. elegans*.

## INTRODUCTION

Alcohol consumption is associated with over 60 diseases, with the risk of these comorbidities generally increasing with greater exposure (*1*). Excessive consumption of alcohol is considered a result of complex interactions between genetic and nongenetic risk factors. Nongenetic factors associated with levels of alcohol intake include gender (*2*), age at first alcohol use (*3*), duration of poverty and involuntary unemployment (*4*), and other lifestyle risk factors (*5*).

Meta-analysis from twin and adoption studies has shown that half of the variance for alcohol use disorder (AUD) is explained by genetic factors (*6*). The discovery of well-replicated risk loci, however, has been limited to the alcohol metabolizing genes alcohol dehydrogenase (*ADH*) and aldehyde dehydrogenase (*ALDH*). Missense variants, rs1229984 (G-->A; p.Arg48His) in *ADH1B* and rs671 (G-->A; p.Glu504Lys) in *ALDH2*, are protective against higher alcohol consumption and alcohol misuse phenotypes (*7*). For example, in a meta-analysis of ~3800 European ancestry individuals, the *ADH1B* rs1229984 variant was strongly associated with reduced risk of alcohol dependence and lower number of maximum drinks in 24 hours (*8*). The *ADH1B* and other *ADH* and *ALDH* variants that are associated with alcohol consumption occur at low frequency among European ancestry populations but are more common in East Asian ancestry populations, where the standardized population prevalence of alcohol misuse is lower (*9*).

Larger samples and genome-wide screens have been used to identify previously unidentified loci beyond the ADH-ADLH cluster. Alcohol consumption phenotypes are of specific interest to the field as they are often more applicable to the wider population than the AUD criteria. Through genome-wide association studies (GWAS), single-nucleotide polymorphisms (SNPs) mapping to/near *KLB*, *AUTS2*, *SERPINC1*, *ANKRD36*, *GCKR*, *PXDN*, *CADM2, HGFAC, SLC39A8*, and *TNFRSF11A* have been associated with alcohol consumption in European ancestry populations at genome-wide significance (*P* < 5 × 10^−8^) (*10*–*15*). However, apart from association signals at *KLB* and *GCKR*, strong evidence of replication has been limited.

In this study, our aim was to determine factors associated with heavy alcohol consumption in white British individuals from the U.K. Biobank (UKB) (www.ukbiobank.ac.uk/), alongside exploring the functional relevance of genome-wide significant variants using model organisms and data mining techniques.

## RESULTS

### Nongenetic factors

The application of the phenotype definition resulted in the identification of 21,967 cases and 103,282 controls that had complete data for all covariates. The covariates included in the final logistic regression model and carried forward to the GWAS analysis were (table S1) age at recruitment, sex, smoking status (anytime versus never), property ownership (own versus rent), body mass index (BMI), Townsend deprivation index at recruitment, adopted as a child, and long-standing illness, disability, or infirmity (yes or no).

### High alcohol consumption loci

**Fig. 1:**
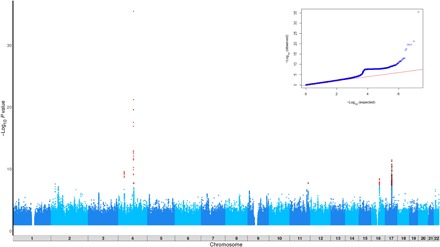
Manhattan plot of the GWAS outcomes for alcohol consumption phenotype using the entire UKB cohort (*n* = 125,249). Inset: QQ plot of expected versus observed GWAS results from UKB, demonstrating modest deviation from the null, λ_GC_ = 1.09.

We tested for SNP-level association with our high alcohol consumption phenotype in UKB. A total of 11,141,077 SNPs survived central quality control (QC) by UKB and post-GWAS filtering for imputation quality and minor allele frequency. The GWAS data test statistics showed modest deviation from the null (λ_GC_ = 1.09; Fig. 1, inset), although linkage disequilibrium (LD) score regression intercept = 1.02 suggests most of the inflation is consistent with polygenic architecture. We then carried forward lead SNPs at *P* < 5 × 10^−6^ from UKB to Genetic Epidemiology Research in Adult Health and Aging (GERA) for replication. We report validated associations that meet genome-wide significance in the meta-analysis of UKB and GERA, which also demonstrate nominal association with the same direction of effect in GERA (Table 1). A summary of all SNPs reaching *P* < 5 × 10^−8^ in UKB can be found in table S2. We identified six loci attaining genome-wide significant association with alcohol consumption after meta-analysis and meeting our criteria for replication: *ADH1B* (rs1229984; *P*_meta = 2.3 × 10^−66^); *KLB* (rs13130794; *P*_meta = 5.7 × 10^−16^); *BTF3P13* (rs144198753; *P*_meta = 4.1 × 10^−29^); *GCKR* (rs1260326; *P*_meta = 1.5 × 10^−13^); *SLC39A8* (rs13107325; *P*_meta = 6.7 × 10^−9^); and *DRD2* (rs11214609; *P*_meta = 4.3 × 10^−9^) (Table 1).

**Table 1:** Summary of validated associations that meet genome-wide significance in the meta-analysis of UKB and GERA, which also demonstrate nominal association with the same direction of effect in GERA. OR, odds ratio; CI, confidence interval.

**UKB heavy alcohol drinker status (cases versus controls)**									**GERA (replication)** **drinks/week (among** **drinkers)**		**Meta-** **analysis**
**Chr**	**Position**	**Lead SNP**	**Locus**	**Risk allele**	**Other allele**	**RAF**	**OR** **(95% CI)**	***P***	**β (SE)**	***P***	***P***
2	27730940	rs1260326	*GCKR*	C	T	0.612	1.06 (1.04–1.08)	2.6 × 10^−8^	0.033 (0.029)	1.1 × 10^−6^	1.5 × 10^−13^
4	39422242	rs13130794	*KLB*	T	C	0.632	1.07 (1.05–1.09)	2.6 × 10^−10^	0.035 (0.007)	4.2 × 10^−7^	5.7 × 10^−16^
4	99713350	rs144198753	*BTF3P13*	C	T	0.991	1.66 (1.48–1.85)	2.70 × 10^−18^	0.156 (0.023)	2.1 × 10^−11^	4.1 × 10^−29^
4	100239319	rs1229984	*ADH1B*	C	T	0.980	1.58 (1.48–1.70)	3.30 × 10^−36^	0.187 (0.016)	2.9 × 10^−32^	2.3 × 10^−66^
4	103188709	rs13107325	*SLC39A8*	C	T	0.928	1.12 (1.08–1.16)	1.60 × 10^−8^	0.029 (0.013)	0.0249	6.7 × 10^−9^
11	113316102	rs11214609	*DRD2*	G	C	0.395	1.06 (1.04–1.08)	2.10 × 10^−7^	0.021 (0.007)	0.0035	4.30 × 10^−9^

#### Multiple distinct signals of association observed at alcohol consumption loci

Conditional analyses revealed an additional signal (*P* < 1 × 10^−5^) (Table 2) at the *SLC39A8* locus (*NFKB1*). Given *ADH1B* and *BTF3P13* are located <1 Mb apart on q23 of chromosome 4, we conducted a wider conditional analysis across a 1.5-Mb region, which included both SNPs. The analysis identified eight independent SNPs mapping to/near *ADH1A*, *ADH1B*, *ADH4*, *ADH5*, *TSPAN5*, and *EIF4E*. The signal mapping to *BTF3P13* did not meet locus-wide significance in conditional analysis, suggesting a false positive for this variant.

**Table 2:** Summary of conditional analysis using outcomes from UKB. Joint models refer to the estimated joint effects of all selected SNPs in a region (i.e., all independent SNPs are fitted together).

**Primary signal**	**Chr**	**SNP**	**Nearest** **gene**	**Position**	**Ref** **allele**	**Ref allele** **frequency**	**β**	**SE**	***P***	**Joint β**	**Joint SE**	**Joint *P***
rs13107325	4	rs13107325	*SLC39A8*	103188709	C	0.928	0.111	0.020	1.6 × 10^−8^	0.112	0.020	1.4 × 10^−8^
rs13107325	4	rs13116422	*NFKB1*	103387160	T	0.916	0.091	0.019	1.9 × 10^−6^	0.092	0.019	1.7 × 10^−6^
**Extended analysis of 4.q23 region containing ADH1B (rs1229984) and BTF3P13 loci (rs144198753)**												
	4	rs1229984	ADH1B	100239319	T	0.020	−0.455	0.036	3.3 × 10^−36^	−0.467	0.036	8.7 × 10^−38^
	4	rs551676206	ADH5	100019089	A	0.990	0.514	0.053	6.2 × 10^−22^	0.528	0.054	6.1 × 10^−23^
	4	rs62307263	ADH1A	100167112	C	0.899	0.083	0.017	1.1 × 10^−6^	0.169	0.023	1.9 × 10^−13^
	4	rs140859990	TSPAN5	99456373	G	0.937	−0.102	0.021	2.0 × 10^−6^	−0.102	0.021	1.9 × 10^−6^
	4	rs182381822	ADH4	100179872	T	0.993	−0.274	0.069	7.2 × 10^−5^	−0.344	0.069	7.1 × 10^−7^
	4	rs1800761	ADH4	100065593	T	0.183	−0.046	0.013	0.00054	−0.401	0.034	5.3 × 10^−33^
	4	rs200411287	ADH4	100078163	A	0.851	−0.023	0.015	0.12	−0.520	0.038	4.4 × 10^−42^
	4	rs150187763	EIF4E	99801016	A	0.982	0.010	0.039	0.79	−0.313	0.047	2.8 × 10^−11^

#### Previously reported loci

The signals described in this section meet our validated association criteria and have been reported for various alcohol phenotypes by other groups. The lead SNP at *ADH1B*, rs1229984 [risk allele frequency (RAF), 0.980; *P* = 3.3 × 10^−36^; fig. S1A], is the missense variant (G-->A; p.His48Arg) that has been widely replicated. The lead SNP rs13130794 (RAF, 0.632; *P* = 4.0 × 10^−9^; fig. S1B) is located in the *KLB* locus and has been reported to be associated with alcohol intake in the UKB (*11*) and a separate European cohort of >98,000 individuals (*10*). The lead variant in chromosome 2, rs1260326 (RAF, 0.612; *P* = 2.6 × 10^−8^; fig. S1C), is in *GCKR*, a glucokinase regulatory gene. This specific SNP has been reported as genome-wide significant for alcohol consumption (i.e., drinks/week) in large-scale European ancestry (*11*, *13*) and transethnic populations (*15*). The lead SNP rs13107325 (RAF, 0.928; *P* = 1.6 × 10^−8^; fig. S1D) is in the zinc transporter gene, *SLC39A8*, which has been linked in Europeans to AUD Identification Test (AUDIT) (*14*) and AUDIT-C outcomes, and to AUD diagnosis (*15*). Last, rs11214609 (RAF, 0.395; *P* = 4.3 × 10^−9^; fig. S1E) was the SNP in the *DRD2* locus. *DRD2* often has been cited in addiction phenotypes and has been identified for AUD, but not alcohol consumption (*15*).

#### Nonreplicated signals reported elsewhere

We also observed genome-wide significant evidence of association in UKB at *FTO* and *CRHR1*, but these signals could not be validated in GERA. There is, however, evidence for association with alcohol-related phenotypes at these loci from other studies. The lead SNP rs55872725 (RAF, 0.599; *P* = 2.6 × 10^−8^) is in the *FTO* gene. This locus has recently been reported to be associated with AUDIT-C and AUD diagnosis in European ancestry individuals (*15*). Different index variants were reported between studies, rs62033408 for AUDIT-C and AUD diagnosis outcomes, but the SNPs are in strong LD with each other (*r*^2^ = 0.92). The *FTO* locus has been strongly associated with BMI, obesity, and, subsequently, type 2 diabetes as a clinical end point. Our lead SNP in this locus is in complete LD (*r*^2^ = 1.0) with rs1558902 in Europeans, which is the lead SNP for BMI in the largest published GWAS to date (*16*). The *CRHR1* locus, with rs1635291 (RAF, 0.754; *P* = 4.5 × 10^−10^) as the lead SNP, has been identified through gene-based analysis in a previous alcohol consumption GWAS where never drinkers were excluded. However, no other groups have reported this locus directly through GWAS. Given the previous associations for these loci with covariates included in our analysis but not in the GERA dataset, we explored the potential for collider bias at rs55872725 when not adjusting for BMI, and rs1635291 when not adjusting for smoking; the results were consistent at 6.5 × 10^−6^ and 2.8 × 10^−8^, respectively. We also found our lead SNP in the *CRHR1* locus to be in strong LD (*r*^2^ = 0.87) with a tag SNP rs1800547 for a common inversion polymorphism in 17q21.31 (*17*).

### In silico analysis

Of the six validated variants from the UKB and GERA cohorts, three were identified as expression quantitative trait loci (eQTLs) through the Genotype-Tissue Expression (GTEx) database (table S3). rs11214609 showed evidence of being an eQTL in various tissues for nearby genes, *ANNK1* and *TTC12*. rs13130794 was associated with the expression of *RFC1* in the cerebellar hemisphere and skeletal muscle and UDGH in blood. rs1260326 was a broader eQTL with evidence across eight loci and various tissues including skeletal muscle, thyroid, and adrenal glands. Table S4 describes the LD between the top eQTL SNP for any eQTL signal and the GWAS SNP. None of the SNP pairs demonstrated evidence of colocalization based on a threshold of LD *r*^2^ > 0.8.

The validated SNPs were submitted to Gene ATLAS to explore phenome-wide association study (PheWAS) outcomes in disease phenotypes via *International Statistical Classification of Diseases, 10th Revision* (*ICD-10*) codes. Evidence suggests that these SNPs contribute to a range of diseases including alcohol dependence, hypertension, skeletal disorders, gout, alcoholic liver disease, ischemic heart diseases, metabolic disorder, obesity, and diabetes mellitus (table S5). The *ADH1B* variant is associated with lipid metabolism disorder, giving further link between alcohol intake and liver fat accumulation.

A set of 37 loci, which reached 5 × 10^−6^ with heavy drinker status phenotype in UKB, were submitted to the Reactome Knowledgebase for pathway analysis (table S6). Six pathways across three distinct processes were found to be significant. The most prominent outcome related to signaling of phosphatidylinositol 3-kinase (PI3K) and PI3K/AKT pathways, particularly in reference to cancer. Dysfunction of the PI3K/AKT pathway is widely implicated in many cancers and is a key regulator of cell survival through downstream targets (*18*). The genes implicated in these pathways were *KLB* and *ESR1* (fig. S2). The other two pathways were neurexins and neuroligins, driven by *LRRTM4* and *NRXN3*, and *TFAP2* (AP-2) family regulation of transcription of growth factors and their receptors, driven by *ESR1*.

**Fig. 2:**
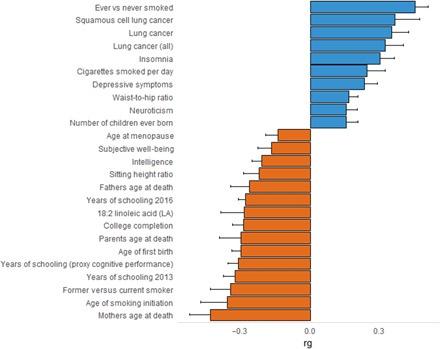
Genetic correlation between alcohol phenotype and other traits using LD score regression from LDHub.

Through genetic correlation analysis of the entire genome, we identified 21 significant correlations that survived multiple testing correction. These outcomes are summarized in Fig. 2. The traits with the strongest correlations included smoking variables [e.g., ever versus never smoked (rg = 0.48, *P*_FDR_ = 2.60 × 10^−13^) and age of smoking initiation (rg = −0.41, *P*_FDR_ = 0.006)], several lung cancer outcomes [e.g., squamous cell lung cancer (*rg* = 0.37, *P*_FDR_ = 0.006) and lung cancer (rg = 0.36, *P*_FDR_ = 1.20 × 10^−4^)], and mothers age at death (rg = −0.41, *P*_FDR_ = 1.60 × 10^−4^). Several education measures and mental health conditions were also found to have significant correlations.

Mendelian randomization (MR) was used to examine the causal relationship between our heavy drinker case-control phenotype and 111 selected traits and clinical outcomes. The number of SNPs used for instrumental variables for each outcome test varied between two and six. Twelve outcomes including four insulin-related and two lung cancer outcomes demonstrated nominal significance using the inverse variance–weighted (IVW) method, although only evidence of a protective effect for ischemic stroke survived multiple testing correction (table S7). The MR-Egger regression intercept demonstrated no evidence of horizontal pleiotropy for the 12 outcomes (*P* ≥ 0.11). Single SNP analysis revealed that rs1229984 was not included in the instrumental variable for ischemic heart disease (SNP or appropriate proxy not available in the outcome dataset). Given rs1229984 demonstrates a consistent and large effect size across genetic studies of alcohol-related phenotypes, it is questionable whether the outcome can be considered as truly representative for this disease.

To further explore the potential causal effect of heavy alcohol consumption on lung cancer outcomes and allow for potential pleiotropy that might be driven by smoking, we repeated our GWAS analysis stratified for smoking status (ever versus never) and performed MR to assess potential collider bias. The SNPs used as the instrumental variable in the original analysis were retained, and lung adenocarcinoma and lung cancer were the only outcomes investigated. Evidence of consistent outcomes was observed in both stratified groups using IVW, although lung cancer in never smokers was the only outcome that did not reach the statistical significance threshold (*P* = 0.085).

### Functional effects in model organisms

**Fig. 3:**
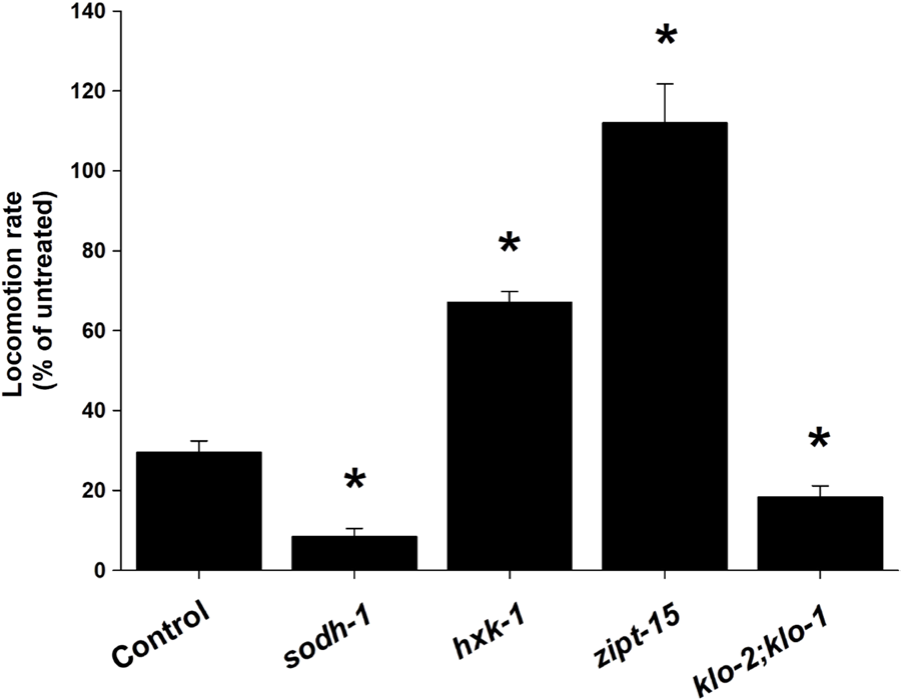
Genetic targets identified by GWAS have conserved roles in alcohol phenotypes. *C. elegans* with loss-of-function mutations in worm orthologs to ADH (*sodh-1*), glucokinase (*hxk-1*), solute carrier family 39 member 8 (*zipt-15*), and β-Klotho protein (*klo-2;klo-1*) were exposed to ethanol, and the resultant effect on locomotion rate was determined. Results are presented normalized to locomotion of untreated worms [basal locomotion rate: 99.03 ± 1.47 (Bristol N2 controls), 103.13 ± 3.66 (*sodh-1*), 87.37 ± 1.91 (*hxk-1*), 31.43 ± 2.97 (*zipt-15*), and 99.90 ± 21.7 (*klo-2;klo-1*)]. *P < 0.05.

To verify whether validated genetic targets (i.e., *ADH1B*, *GCKR*, *SLC39A8*, and *KLB*) had a conserved role in phenotypic responses to alcohol, we investigated the acute effects of ethanol on the nematode worm, *Caenorhabditis elegans*. In comparison to wild-type animals, those with a loss-of-function mutation in the worm ortholog for *ADH* (*sodh-1* in *C .elegans*) had a statistically enhanced ethanol response (Fig. 3) as has been previously described in detail (*19*). The effect of intoxicating ethanol on coordinated locomotion was next quantified for loss-of-function mutations in *C. elegans* glucokinase (GK; *hxk-1*) and solute carrier family 39 member 8 (*SLC39A8*; *zipt-15*) (Fig. 3). Without an ortholog for GCKR in *C. elegans*, we instead analyzed its downstream effector protein glucokinase itself. Loss-of-function mutations in these genes significantly reduced the effect of ethanol for GK and *SLC39A8* (Fig. 3), underlining a conserved role for these genes in whole-animal responses to alcohol. We also quantified single mutations in the *C. elegans* orthologs for the β-Klotho protein (*KLB*; *klo-1* and *klo-2*) and found that individual mutations did not alter the ethanol phenotype (fig. S3A). A compound mutation of both *klo-1* and *klo-2* (*20*), however, did have a significantly enhanced ethanol effect (Fig. 3).

**Fig. 4:**
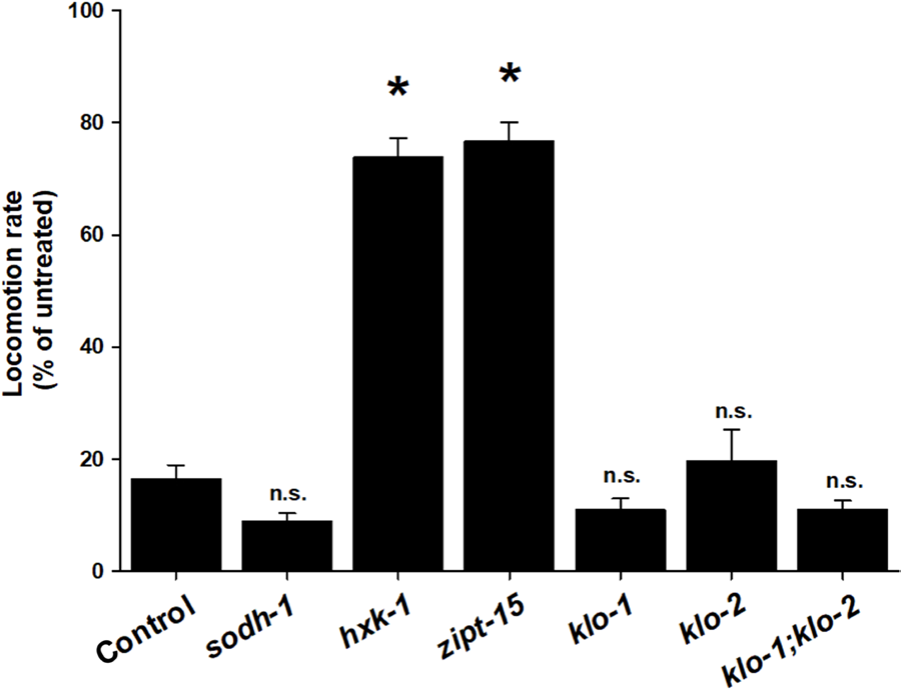
RNAi of genetic targets. RNAi knockdown of worm orthologs to glucokinase (*hxk-1*) and solute carrier family 39 member 8 (*zipt-15*) phenocopies the loss-of-function mutations. Results are presented as locomotion of worms treated with ethanol normalized to untreated worms [basal locomotion rate: 87.63 ± 21.6 (empty vector control), 94.17 ± 2.91 (*sodh-1*), 77.60 ± 2.34 (*hxk-1*), 60.0 ± 2.34 (*zipt-15*), 90.97 ± 3.56 (*klo-1*), 99.13 ± 2.78 (*klo-2*), and 110.0 ± 3.40 (*klo-2;klo-1*)]. *P < 0.05; n.s., not significant.

To validate the effects seen in individual mutant strains, we performed RNA interference (RNAi) experiments to knock down expression of the contraindicated genes. In comparison to control, RNAi knockdown of GK (*hxk-1*) and *SLC39A8* (*zipt-15*) resulted in the same phenotypic effects as did the mutations (Fig. 4). In our RNAi experiments, knockdown of *ADH* (*sodh-1*) did not result in a significant decrease. Similar to the *KLB* mutations, individual knockdown of *C. elegans KLB* (*klo-1* or *klo-2*) did not statistically enhance the ethanol phenotype and neither did knocking down both *klo-1* and *klo-2* simultaneously (Fig. 4). The lack of effect in the double knockdown is perhaps expected given that RNAi efficiency can be reduced with multiple targets (*21*). To validate the alcohol effect of *KLB* in *C. elegans* in an alternative method, we performed RNAi on individual *KLB* genes in the mutant strain of the other ortholog (i.e., *klo-1* RNAi in the *klo-2* background; *klo-2* RNAi in the *klo-1* background). In both cases, there were exceptionally enhanced effects of ethanol similar to that seen with the compound mutant strain (fig. S3B).

## DISCUSSION

We report here a large alcohol consumption GWAS, including 125,249 white British participants, with subsequent replication and meta-analysis in an additional 47,967 individuals. Moreover, and as promoted by Salvatore and colleagues in this field (*22*), we conducted a post-GWAS study to investigate the biological implications of our findings. This includes providing evidence of a conserved role in phenotypic responses to alcohol for all targets available for investigation (*ADH1B*, *GCKR*, *SLC39A8*, and *KLB*) in *C. elegans*.

The primary strengths of this study are the (i) large sample size; (ii) replication and subsequent meta-analysis; (iii) post-GWAS analysis, including functional assessment using *C. elegans*; and (iv) use of a mixed-model approach in GWAS to account for relatedness. There are, however, several limitations that require discussion. First, the alcohol data and, therefore, the case-control phenotypes are based on self-reported alcohol intake. It is well documented that individuals underreport their alcohol consumption for a number of reasons. This presents risk of cases being mislabeled as controls, alongside the granularity of the data being reduced by the categorical approach. There are also differences in the measurement scale between discovery and replication cohorts. This difference was handled by applying a *z* score approach to meta-analysis. Second, we restricted analysis to those of white British ancestry to limit population structure variability on outcomes. This restricts generalizability outside of European populations. Third, we recognize limitations to our MR approach: (i) MR is considered most powerful when instrumental variables are from a continuous trait. This is of greater concern, however, when a disease-specific phenotype is used for instrument selection because of the likely contribution of various factors in disease pathology; and (ii) an inherent assumption of MR is that variants show no pleiotropy or direct effects on the outcome. This requires knowledge of the underlying biology under investigation, although this is rarely complete. Last, we were unable to undertake functional assessment of all genome-wide significant loci due to there being either no specific *C. elegans* orthologs, or too many nonspecific orthologs, or fatal consequences of gene knockdown.

The largest and most robust effects were observed in *ADH1B*, including replicated findings from the work in *C. elegans* for *ADH* (*19*), providing confidence for the selected phenotype. The biological validity of polymorphisms in *ADH* loci is well documented and discussed in detail in other GWAS publications (*12*).

*KLB* has been previously associated with alcohol phenotypes in European populations (*10*, *12*). A biological basis for *KLB* has been proposed in mice, where those lacking β-Klotho had increased alcohol consumption (*10*). This behavior was refractory to recombinant fibroblast growth factor 21 (FGF21), a hormone involved in sugar intake regulation and for which β-Klotho is an obligate coreceptor. Hence, down-regulation of *KLB* may lead to sustained intake of alcohol and/or high-sugar food. Moreover, loss of both *KLB* isoforms in *C. elegans* caused an enhancement in the ethanol effects. Further evidence for energy processing pathways being implicated in alcohol consumption is demonstrated by the genome-wide significant outcomes for *GCKR* and *SLC39A8*, with these findings being consistent with recent publications (*11*, *13*, *14*). The data from our functional work in hexokinase and ZRT/IRT-like protein transporter supports the role of glucose metabolism pathways in the susceptibility to heavy alcohol consumption by demonstrating attenuation of the depressive effects of high-dose alcohol when *hxk-1* and *zipt-15* are independently knocked down. Although we failed to demonstrate replication between the UKB and GERA cohorts, potentially due to variation in phenotype, evidence from other GWAS showed consistent effects for *FTO* (*23*). The suggestive association with this pleiotropic locus adds further plausibility of common pathways implicated in the consumption of food and alcohol. The purported shared pathogenic architecture may result in dysregulation of brain reward pathways leading to excess consumption (*24*). Controlling for BMI within our GWAS suggests that the associations for alcohol consumption are independent of BMI, adding weight to the hypothesis of a potentially shared, rather than mediated, pathways.

*DRD2* encodes the dopamine receptor 2 subtype and is linked to several neurobiological processes, including functional activation of reward circuits (*25*). Data from in vivo and in vitro experiments show *DRD2* to be a susceptibility gene for alcohol dependence (*26*), and altering *DRD2* expression leads to differential responses to substances and stimuli (*27*), conferring increased risk for addiction. Moreover, evidence suggests increased risk of relapse in alcohol and cocaine dependence, and heightened heroin, nicotine, and glucose craving when polymorphisms of *DRD2* are present or there is low D2 receptor availability (*28*). The association of *DRD2* with alcohol was confirmed in GWAS findings for AUD but not alcohol consumption, with authors proposing that the central nervous system is a fundamental element in the progression to clinical diagnosis (*15*). Our findings are somewhat contradictory given that participant categorization is based on U.K. alcohol units consumed per week, although the quantities for cases are often associated with high risk of AUD.

Together, the loci outside of the *ADH/ALDH* cluster suggest several common pathways associated with different types of compulsive behavior and addiction phenotypes. Considerable evidence from animal models and from humans supports convergence of these common etiologies in the brain’s limbic system regardless of the prior distinct mechanism of action and ultimate observable phenotype (*29*, *30*). This suggests that addiction might be better considered as a pathobiological risk with different endotypes, rather than each specific phenotype (e.g., alcohol dependence, drug addiction, and gambling addiction) being independently characterized. From a therapeutics perspective, these outcomes provide additional and supportive evidence toward a number of targets that might be amendable to pharmacological intervention. Further investigation is required to determine which sites have the greatest potential. Data from the Open Targets resource (www.opentargets.org/) suggest that 49 drugs have reached phase IV investigation for *DRD2* across a range of indications, including mental health disorders and cocaine dependence; no drugs are in development for *ADH1B*, *KLB*, *GCKR*, or *SLC39A8*. *FGF21* has been explored due to links with *KLB*, but no drugs are in the market yet.

Using the GWAS outcomes from UKB enabled us to examine the relations between key variants/loci and traits and disease phenotypes. Genetic correlation analysis and MR consistently demonstrated an association with lung cancer. Determining alcohol’s contribution to lung cancer often has been limited by the strong positive correlation between alcohol intake and smoking. However, the outcomes from the MR provide potential evidence of a causal relationship in our overall sample and when stratified by smoking status. Alcohol is a known carcinogen and is implicated in cancers of the liver, colon, rectum, head and neck, and breast, for example (*31*), while evidence for lung is variable (*32*, *33*). Lung cancer is a complex and multifactorial disease involving genetic and a range of measurable and nonmeasurable environmental and lifestyle factors. Hence, heavy alcohol consumption is one potentially modifiable risk factor to reduce disease incidence. An alternative hypothesis is through a joint risk locus in *KLB* that independently drives alcohol consumption and cancer risk. In addition to the above, β-Klotho inhibits PI3K and, subsequently, AKT, an important pathway in normal cell function. The dysfunction of the PI3K/AKT pathway, identified in our pathway analysis, has been cited in cancerous cells and as a risk factor in cancer onset (*18*, *34*). Down-regulation of *KLB* has been reported across several cancers (*35*, *36*). However, some variations in findings exist (*37*), and no evidence is available in lung cancer. Basic cell line study would provide initial data on β-Klotho expression in lung tumor cells.

Links to other diseases were also found. Drinking heavily was suggested as a protective factor for ischemic stroke. This is not consistent with traditional epidemiological findings or other MR findings using rs1229984 as the instrumental variable (*38*). However, the lack of rs1229984 in our instrumental variable for this analysis means the outcome should be interpreted with caution. The nominal evidence in several insulin measures suggests a wider biological association with glucose regulation, linking back to the potential importance of energy metabolism pathways in alcohol consumption. *ADH1B* and *GCKR* were associated with gout, and *ADH1B* alone with hypertension. The lead SNP at *GCKR*, rs1260326, has been shown to be a risk variant for gout in a separate GWAS (*39*), and rs1229984 in *ADH1B* has been identified for systolic blood pressure using a functional enrichment approach. Increasing alcohol consumption is a known risk factor for both gout and hypertension (*40*). Last, there was evidence for several skeletal complications with identified alcohol consumption variants. Alcohol intake represents a dose-dependent risk factor for fragility fractures due to the direct effects of alcohol on bone cell metabolism. Chronic alcohol consumption has been associated with a twofold increased risk of hip fracture in prospective cohort studies involving more than 16,000 subjects (*41*).

## CONCLUSION

Our findings offer insight into genes, pathways, and causal relationships for disease risk associated with heavy alcohol consumption. The inclusion of model organism work to investigate the conserved role of loci alongside GWAS outcomes is novel in the alcohol field and adds validity for relevant outcomes. In addition, the correlation between the *C. elegans* phenotypic data with genome-wide association in humans reinforces a link between the acute physiological effect of alcohol and predisposition to excessive alcohol consumption. Specific findings suggesting joint reward/addiction pathways, the role of energy metabolism, casual links to lung cancer, and shared genetic architecture with gout and hypertension are of particular interest. Further investigation is required, however, to realize the potential of these outcomes and result in meaningful population—or clinical-level impact.

## MATERIALS AND METHODS

### Discovery cohort

UKB is a large population cohort of ~502,000 individuals from the United Kingdom aged 40 to 69 years at the time of recruitment. Baseline assessment was undertaken at one of 22 centers across the United Kingdom between 2006 and 2010. Each participant completed a comprehensive demographic, lifestyle, and health questionnaire, underwent clinical phenotyping, provided biological samples (i.e., blood, urine, and saliva), and agreed to have his or her health record accessed for baseline and follow-up outcomes (*42*). Ethical approval for UKB was gained from the Research Ethics Service (REC reference: 16/NW/0274), and written informed consent was obtained from all participants. The current analyses were conducted under approved UKB data application number 15110.

#### Phenotype definition

Questions from the UKB baseline assessment were used to develop two study groups: heavy drinkers (cases) and drinkers not reaching criteria for cases (controls). All participants who indicated they consumed alcohol were asked to quantify their intake per week or per month using standard drink sizes [e.g., “In an average WEEK, how many glasses of RED wine would you drink? (there are six glasses in an average bottle)”]; pictures accompanied these questions to provide visual representation of each measure. We then applied a standardized number of U.K. alcohol units to each drink to enable an estimated number of units per week to be calculated (see the Supplementary Materials for more detail). Sex-specific heavy drinking was then defined as >35 U/week for women and >50 U/week for men. Any cases with values >4 SDs above the gender-specific means were removed. Controls were individuals who were not current abstainers from alcohol (i.e., ≥1 U/week) but did not reach the sex-specific criteria for heavy drinking and were drinking at similar levels to 10 years previous.

#### Genotyping, imputation, QC, and GWAS

In July 2017, UKB released genetic information (directly typed and imputed genotypes) for the entire cohort (*n* = 487,406) to approved collaborators. Most (90%) of the participants were genotyped on the UKB Axiom Array, with the remaining 10% genotyped on the Affymetrix UK BiLEVE Axiom Array. There is >95% content overlap between arrays. Genotyping, QC, and imputation were performed centrally by UKB and has been previously described (*43*). Imputation was performed up to combined reference panels from the 1000 Genomes Project (Phase 3), UK10K, and Haplotype Reference Consortium (*44*). Analyses were restricted to a subset of white British individuals, defined on the basis of self-reported ethnicity and genetic data.

Using UKB data, univariate and multivariate logistic regressions were used to determine covariates to be included in the GWAS analysis. Variables only available for the entire cohort and implicated in previous research were considered, and any values >4 SDs from the mean were removed (*n* = 7649 participants removed due to missing data). Variables reaching *P* < 0.01 in separate univariate analysis were carried forward to a multivariate model, where the stepAIC function of the “MASS” R package was used to determine stepwise entry of variables into the model. Collinearity was determined using variance inflation factor, and variables were accordingly removed from the final model (Variance Inflation Factor >10). An a priori decision was made to include age and sex in all models. Our rationale for using this approach is twofold: first, to account for confounding factors that may bias effect estimates, and second, to improve power by reducing residual variance.

Genetic association analysis in autosomes was conducted using a linear mixed model in BOLT-LMM v2.3.1 (*45*), adjusted for age at recruitment, sex, genotyping array, and nongenetic covariates identified in the logistic regression model. The BOLT-LMM model includes a random effect derived from a genetic relationship matrix to account for population structure and relatedness. Potential *P* value inflation due to residual population structure and relatedness was checked using genomic control following filtering of variants based on imputation quality (INFO ≥0.4) and minor allele frequency of 0.005. Distance-based clustering was used for defining loci, such that genome-wide significant SNPs were ranked from most significant to least significant, and SNPs were retained if they did not map ±500 kb of a more significant SNP. Variants reaching *P* < 5 × 10^−6^ and surviving distance-based clustering (i.e., lead SNPs) in the UKB cohort were explored in the GERA cohort for the purposes of replication.

### Replication cohort

The GERA cohort was used for replication. GERA is part of the Kaiser Permanente Research Program on Genes, Environment, and Health (RPGEH) and has been described in detail elsewhere (*46*). In short, the cohort comprises 110,266 adult men and women who are consented participants in the RPGEH, an unselected cohort of adult participants who are members of Kaiser Permanente Northern California, an integrated health care delivery system. All study procedures were approved by the Institutional Review Board of the Kaiser Foundation Research Institute.

For this replication analysis, 47,967 GERA participants of non-Hispanic white ethnicity who had alcohol consumption information were included. Alcoholic drinks consumed per week as a quantitative trait (drinks/week) was assessed on the basis of the RPGEH survey as previously described (*12*) and as part of the Supplementary Materials. Genotyping using Affymetrix Axiom arrays (Affymetrix, Santa Clara, CA, USA) (*47*, *48*), imputation using the cosmopolitan 1000 Genomes Project reference panel, and GWAS analysis were undertaken as detailed in the Supplementary Materials.

### Meta-analysis

METAL was used to perform a fixed-effects meta-analysis between the UKB and GERA cohorts using Stouffer’s method to account for the effect sizes in discovery and replication being on different scales (*49*). An overall *z*-statistic and *P* value were calculated from a weighted sum of the individual statistics. Weights are proportional to the square root of the number of individuals examined in each sample and selected such that the squared weights sum to 1.0.

A validated association was defined as follows: (i) reaching *P* < 5 × 10^−6^ in the discovery cohort, (ii) demonstrating nominal association with the same direction of effect in the replication cohort, and (iii) meeting genome-wide significance in the meta-analysis of both datasets sets.

### Conditional analysis

Conditional analysis was performed on validated associations using Genome-wide Complex Trait Analysis (*50*) (http://cnsgenomics.com/software/gcta/) and the GWAS outcomes from the UKB to identify independent signals in the same region as each lead SNP (±500 kb); one model was fitted per region. A set of 5000 randomly selected UKB white British participants was used to develop a reference set to approximate LD. A threshold of *P* < 1 × 10^−5^ was used to select index SNPs for independent signals in each region, where the conditional estimates were derived from fitting all independent SNPs jointly (i.e., joint model).

### In silico analysis

#### Expression quantitative trait loci

The GTEx Portal (http://www.gtexportal.org) was used to assess whether the lead SNP at each locus was an eQTL for local genes across the range of available tissues (*51*). This approach uses gene expression information across various human tissue types and genotype data to build information on eQTLs using a ±1-Mb cis-window around the transcription start site. All tissue types with more than 70 samples available within GTEx were evaluated in our analysis including the brain, heart, liver, skeletal muscle, and skin. Significant eQTLs were based on a false discovery rate (FDR < 0.05) correction. The LD between the top eQTL SNP for any eQTL signal and the GWAS SNP was assessed to explore whether the two signals colocalize with each other; an LD *r*^2^ > 0.8 in Europeans from the 1000 Genomes was considered evidence of colocalization.

#### Genetic correlations

LD Hub v1.9.0 (http://ldsc.broadinstitute.org/ldhub/) was used to identify genetic correlations through LD score regression between the binary alcohol phenotype and other complex traits (*52*). This method uses individual SNP allele effect sizes from GWAS summary statistics and the average LD in a region to estimate bivariate genetic correlations. We tested for genetic overlap between alcohol consumption from our GWAS and disease outcomes and related traits in European cohorts available in the LD Hub, except for UKB outcomes and metabolites due to the large number of potential comparisons. FDR < 0.05 was used to account for multiple comparisons.

#### Mendelian randomization

MR-Base v0.4.21 was used for performing two-sample MR to explore the causal relationship between alcohol consumption and other disease outcomes and related traits (*53*). Outcomes were selected from the NHGRI-EBI GWAS catalog and filtered for European ancestry–only populations. All genome-wide significant SNPs were initially considered. Before MR analysis, the identified SNPs were explored for independence using estimated LD scores from the 1000 Genomes Project European sample, where *r*^2^ ≥ 0.001 among SNPs in a 10,000-kb region resulted in only the SNP with the lowest *P* value being retained. One hundred eleven outcomes were selected on the basis of being diseases of interest, metabolites influenced by alcohol and prominent in subsequent alcohol-related disease onset or progression (e.g., triglycerides), or other consequences of heavy alcohol consumption. Harmonization between exposure data and outcome data was undertaken to ensure effects corresponded to the same allele. Causal estimates between exposure and outcomes were obtained using the two-sample MR IVW method with FDR for multiple comparisons. Sensitivity analyses to account for pleiotropy were performed using MR-Egger regression and weighted median approaches. The weighted median test has been suggested as an alternative to the MR-Egger when the instrumental variable contains a small number of SNPs.

#### PheWAS

Gene ATLAS (http://geneatlas.roslin.ed.ac.uk/) was used as a lookup for outcomes from PheWAS analysis performed on UKB traits (*54*). The database contains data from >450,000 white British individuals, >31 million variants, and 778 traits; only *ICD-10* traits were considered (*n* = 496). This information was used to derive a phenome-wide significance threshold, α divided by the number of independent tests, i.e., 1.68 × 10^−5^ [0.05/(496*6)].

#### Pathway analysis

Reactome pathway knowledgebase (https://reactome.org/) was used to undertake pathway analysis (*55*). The Reactome Knowledgebase systematically links human proteins to their molecular functions, providing a resource that operates both as an archive of biological processes and as a tool for discovering unexpected functional relationships. Loci identified through distance-based clustering at a relaxed threshold of *P* < 5 × 10^−6^ from the GWAS analysis were included. These loci were mapped to pathways, and a *P* value was calculated on the basis of the overlap between the query and the pathway expression; an FDR correction was applied by the software.

### Assessment of function using model organisms

*C. elegans* is an excellent genetic model for investigating whole-animal effects of alcohol (*56*–*58*). Similar to humans, acute exposure to intoxicating alcohol induces a dose-dependent reduction in coordinated movement of *C. elegans* both in solution (*59*) and on solid agar (*60*). Strains of *C. elegans* were selected on the basis of the outcomes from the present GWAS at the level of *P* < 5 × 10^−8^ and having evidence of replication in GERA or being reported as genome-wide significant in other alcohol phenotype studies.

Phenotypic and RNA interference experiments were performed at 20°C in a temperature-controlled room on young adult hermaphrodites selected from sparsely populated NGM (nematode growth media) plates. As we and others have previously demonstrated (*59*, *60*), exposure to 400 mM external ethanol reduces coordinated locomotion of wild-type (Bristol N2) animals by ~70%. An external concentration of 400 mM ethanol is equivalent to an internal concentration of ~20 to 70 mM, which is equivalent to a blood alcohol level of ~0.1 to 0.4% and is consistent with levels of intoxication experienced by humans. Locomotion rate was the outcome of interest and was quantified by thrashing in Dent’s solution [140 mM NaCl, 6 mM KCl, 1 mM CaCl_2_, 1 mM MgCl_2_, and 5 mM Hepes (pH 7.4) with bovine serum albumin at 0.1 mg/ml] as previous described (*59*, *60*). See the Supplementary Materials for full details.

All functional data are expressed as means ± SE. Thirty treated and untreated animals were analyzed and compared per strain per experiment. Statistical significance was assessed by one-way analysis of variance (ANOVA) with post hoc Bonferroni correction for multiple comparisons.

## Supplementary Material

http://advances.sciencemag.org/cgi/content/full/6/3/eaay5034/DC1

Download PDF

Table S3

Functional validity, role, and implications of heavy alcohol consumption genetic loci

## SUPPLEMENTARY MATERIALS

Supplementary material for this article is available at http://advances.sciencemag.org/cgi/content/full/6/3/eaay5034/DC1 Supplementary Methods Table S1. Summary of final multivariable logistic regression model. Table S2. Summary of genome-wide significant SNPs following distance-based clumping on the UKB cohort, and the replication cohort and meta-analysis outcomes. Table S3. eQTL analysis outcomes. Table S4. LD between the top eQTL SNP for any eQTL signal and the GWAS SNP. Table S5. Variant-trait significant outcomes from PheWAS. Table S6. Variants at 5 × 10^−6^ and submitted to the Reactome Knowledgebase. Table S7. Mendelian randomization results for nominally significant outcomes in the IVW analysis and FDR outcomes using the IVW method. Fig. S1. LocusZoom plots for lead SNPs from GWAS on alcohol phenotype in the entire cohort. Fig. S2. Constitutive signaling by aberrant PI3K in cancer. Fig. S3. Individual *C. elegans* b-Klotho genes outcomes. References (*61*–*71*)

## Appendix

View/request a protocol for this paper from *Bio-protocol*.
