# Supplementary material for: Functional validity, role, and implications of heavy alcohol consumption genetic loci
Source: Sci Adv. 2020 Jan 15;6(3):eaay5034. doi: 10.1126/sciadv.aay5034 (PMC6962045; doi:10.1126/sciadv.aay5034)
Supplement: http://advances.sciencemag.org/cgi/content/full/6/3/eaay5034/DC1 [file supp_6_3_eaay5034__index.html]

Science Advances | Science AdvancesAAASSearchScience AdvancesMenu

## Supplementary Materials

**The PDFset includes:**

- Supplementary Methods
- Table S1. Summary of final multivariable logistic regression model.
- Table S2. Summary of genome-wide significant SNPs following distance-based clumping on the UKB cohort, and the replication cohort and meta-analysis outcomes.
- Legend for table S3
- Table S4. LD between the top eQTL SNP for any eQTL signal and the GWAS SNP.
- Table S5. Variant-trait significant outcomes from PheWAS.
- Table S6. Variants at 5 × 10^−6^ and submitted to the Reactome Knowledgebase.
- Table S7. Mendelian randomization results for nominally significant outcomes in the IVW analysis and FDR outcomes using the IVW method.
- Fig. S1. LocusZoom plots for lead SNPs from GWAS on alcohol phenotype in the entire cohort.
- Fig. S2. Constitutive signaling by aberrant PI3K in cancer.
- Fig. S3. Individual *C. elegans* β-Klotho genes outcomes.
- References (*61*–*71*)

Download PDF

**Other Supplementary Material for this manuscript includes the following:**

- Table S3 (.csv format). eQTL analysis outcomes.

**Files in this Data Supplement:**

- Adobe PDF - aay5034\_SM.pdf
