# Supplementary material for: Functional validity, role, and implications of heavy alcohol consumption genetic loci
Source: Sci Adv. 2020 Jan 15;6(3):eaay5034. doi: 10.1126/sciadv.aay5034 (PMC6962045; doi:10.1126/sciadv.aay5034)
Supplement: Download PDF [file aay5034_SM.pdf]

Science Advances
AAAS

advances.sciencemag.org/cgi/content/full/6/3/eaay5034/DC1

Supplementary Materials for

Functional validity, role, and implications of heavy alcohol consumption genetic loci

Andrew Thompson*, James Cook, Hélène Choquet, Eric Jorgenson, Jie Yin, Tarja Kinnunen, Jeff Barclay, Andrew P. Morris, Munir Pirmohamed

*Corresponding author. Email: andrew.thompson@liverpool.ac.uk

Published 15 January 2020, *Sci. Adv.* **6**, eaay5034 (2020)
DOI: 10.1126/sciadv.aay5034

The PDF file includes:

Supplementary Methods
Table S1. Summary of final multivariable logistic regression model.
Table S2. Summary of genome-wide significant SNPs following distance-based clumping on the UKB cohort, and the replication cohort and meta-analysis outcomes.
Legend for table S3
Table S4. LD between the top eQTL SNP for any eQTL signal and the GWAS SNP.
Table S5. Variant-trait significant outcomes from PheWAS.
Table S6. Variants at 5×10^{-6} and submitted to the Reactome Knowledgebase.
Table S7. Mendelian randomization results for nominally significant outcomes in the IVW analysis and FDR outcomes using the IVW method.
Fig. S1. LocusZoom plots for lead SNPs from GWAS on alcohol phenotype in the entire cohort.
Fig. S2. Constitutive signaling by aberrant PI3K in cancer.
Fig. S3. Individual *C. elegans* β-Klotho genes outcomes.
References (*61–71*)

Other Supplementary Material for this manuscript includes the following:

(available at advances.sciencemag.org/cgi/content/full/6/3/eaay5034/DC1)

Table S3 (.csv format). eQTL analysis outcomes.

Supplementry Methods

UK alcohol units applied to each alcoholic drink in Uk Biobank cohort

The number of units in each type of alcoholic drink was standardised using drink measure (provided by UK) and alcohol by volume data from various sources. Units were determined by multiplying the volume of the drink (in millilitres) by its percentage ABV, and dividing the outcome by 1000.

The units assigned to each drink (Beer/cider = 2.6; White wine = 1.5; Red wine = 1.5; Fortified wine = 1.1; Spirits = 1; Other =1.5) were mutlipled by the self-reported number of specific drinks consumed per week or per month and then summed across all types of drinks. Where information was only available on alcohol consumed per month, the summed data was multipled by 12 (for months in a year) and then divided by 52 to obtain a measure comparabile with weekly alcohol intake.

Replication phenotype and genetic analysis

Phenotype: On the Research Program on Genes, Environment, and Health (RPGEH) survey, participants were asked regarding the past year: "On average, how many days a week do you have a drink containing alcohol?" (no days, 1 day, 2 days, 3 days, 4 days, 5 days, 6 days, or every day). Participants were also asked: "On a typical day that you drink, how many drinks do you have?" (none, 1, 2, 3, 4, 5, 6, 7, or ≥8). The regular quantity of alcohol drinks consumed per week was calculated for alcohol drinkers by multiplying the two answers. This drinks/week quantitative trait was then log-transformed prior to conducting genetic association analyses to address non-normality.

Genotyping: DNA samples from GERA individuals were extracted from Oragene kits (DNA Genotek Inc., Ottawa, ON, Canada) at KPNC and genotyped at the Genomics Core Facility of the University of California, San Francisco (UCSF). DNA samples were genotyped at over 665,000 single nucleotide polymorphisms (SNPs) on Affymetrix Axiom arrays (Affymetrix, Santa Clara, CA, USA) (*47, 48*). Genetic variants with initial genotyping call rate ≥97%, allele frequency difference ≤0.15 between males and females for autosomal markers, and genotype concordance rate >0.75 across duplicate samples were included (*46*). Around 94% of samples and more than 98% of genetic markers assayed passed quality control (QC) procedures. In addition to those QC criteria, SNPs with genotype call rates <90% were removed, as well as SNPs with a minor allele frequency (MAF) < 1%.

Imputation: Following genotyping QC, we conducted statistical imputation of additional genetic variants. Following the pre-phasing of genotypes with Shape-IT v2.5 (*61*), variants were imputed from the cosmopolitan 1000 Genomes Project reference panel (phase I integrated release; http://1000genomes.org) using IMPUTE2 v2.3.1 (*62*). As a QC metric, we used the info r^2 from IMPUTE2, which is an estimate of the correlation of the imputed genotype to the true genotype (*63*). Imputed genetic variants with info-metric $r^2 \geq 0.9$ and MAF≥1% were reported as previously described (*12*).

GWAS Analysis. Analyses were conducted using PLINK (*64*) v1.07 and R. A linear regression of each individual's drinks/week was performed with the following covariates: age at survey, sex, the top 10 ancestry principal components (PCs), and the percentage of Ashkenazi ancestry, as previously described (*12*). Eigenstrat (*65*) v4.2 was used to calculate the PCs (*66*). A linear regression of the

residuals on each SNP was then performed to assess genetic associations with drinks/week. Data from each SNP were modelled using additive dosages to account for the uncertainty of imputation (*67*).

Model Oragnisms – complete methods

Nematode strains and culture

Caenorhabditis elegans strains were grown at 20°C on nematode growth medium (NGM) agar plates using *Escherichia coli OP50* as a food source (*68*). The following strains were used in this study: Bristol N2 (wild-type), *K12G11.3 sodh-1 (ok2799)*, *FX04174 hxk-1 (tm4174)*, *TV13570 nrx-1 (wy778)*, *VC1881 zipt-15 (ok2160)*, *VC2175 klo-1 (ok2925)*, *RB1549 klo-2 (ok1862)*, *TC446;klo-2 (ok1862)*; *klo-1 (ok2925) (20)* and *NL2099 rrf-3 (pk1426)*.

Behavioural assays

Phenotypic experiments were performed at 20°C in a temperature-controlled room on young adult hermaphrodites selected from sparsely populated NGM plates. Locomotion rate was quantified by thrashing in Dent's solution (140 mM NaCl, 6 mM KCl, 1 mM $CaCl_2$, 1 mM $MgCl_2$, 5 mM HEPES, pH 7.4 with bovine serum albumin at 0.1 mg/ml) as previous described (*59, 60*). One thrash was defined as one complete movement from maximum to minimum amplitude and back. Ethanol was mixed with Dent's solution to produce the indicated concentrations. Phenotypic effects of ethanol were calculated as thrashes per minute following a 10 minute exposure and normalised as a percentage of mean thrashing rate of untreated worms measured each day. All data are expressed as mean ± S.E. 30 treated and untreated animals were analysed and compared per strain per experiment. Significance was assessed by one-way analysis of variance (ANOVA) with post-hoc Bonferroni correction for multiple comparisons.

RNA interference

RNA interference experiments were performed on the *rrf-3 (pk1426)* strain as previous described (*60, 69*). RNAi was achieved by feeding (*70*) using the ORFeome based RNAi library (*71*). In brief, the indicated HT115 RNAi bacterial clones were cultured in LB media with 100 µg/ml ampicillin and then spotted in three 50 µl drops on 60 mm diameter NGM plates containing 1 mM isopropyl β-1-thiogalactopyranoside (IPTG) and 25 µg/ml carbenicillin. RNAi plates were dried for at least 4 days before seeding to improve RNAi efficiency. Five L3-L4 worms were added to each RNAi plate and cultured at 20°C. Phenotypic analysis was performed on first-generation progeny fed with the indicated RNAi bacterial clones. For simultaneous RNAi of *klo-1* and *klo-2*, worms were fed both RNAi bacterial clones mixed at equal bacterial concentration quantified by OD600 absorbance. Phenotypic effects of ethanol were quantified as above and compared to RNAi feeding with an empty feeding vector. 30 treated and untreated animals were analysed and compared per strain per experiment.

Table S1. Summary of final multivariable logistic regression model.

Risk Factor	OR	95% CI	*P*
Sex	2.50	2.42 – 2.59	<0.0005
Age at Recruitment	0.96	0.96 – 0.96	<0.0005
Property ownership (own vs. rent)	1.53	1.45 – 1.63	<0.0005
Smoking status (anytime vs. never)	3.22	3.11 – 3.32	<0.0005
Long-standing illness, disability or infirmity (yes vs. no)	1.19	1.15 – 1.23	<0.0005
Townsend deprivation index at recruitment	1.09	1.08 – 1.10	<0.0005
Adopted as a child (yes vs. no)	1.16	1.02 – 1.33	0.024
BMI	1.06	1.06 – 1.06	<0.0005

Table S2. Summary of genome-wide significant SNPs following distance-based clumping on the UKB cohort, and the replication cohort and meta-analysis outcomes.

UK Biobank									GERA (replication)		Meta-analysis	
**Chr**	**Position**	**Lead SNP**	**Locus**	**Risk Allele**	**Other Allele**	**RAF**	**OR (95%CI)**	**P**	**BETA (SE)**	**P**	**Z-score**	**P**
2	27730940	rs1260326	*GCKR*	C	T	0.612	1.06 (1.04-1.08)	2.60E-08	0.033 (0.029)	1.12E-06	7.391	1.46E-13
4	39422242	rs13130794	*KLB*	T	C	0.632	1.07 (1.05-1.09)	2.60E-10	0.035 (0.007)	4.20E-07	8.096	5.67E-16
4	99713350	rs144198753	*BTF3P13*	C	T	0.991	1.66 (1.48-1.85)	2.70E-18	0.156 (0.023)	2.12E-11	10.994	4.10E-29
4	100239319	rs1229984	*ADH1B*	C	T	0.980	1.58 (1.48-1.70)	3.30E-36	0.187 (0.016)	2.91E-32	17.209	2.27E-66
4	102456330	rs76062146	*BANK1*	G	A	0.972	1.19 (1.12-1.27)	1.90E-08	0.027 (0.020)	0.1866	5.193	2.07E-07
4	103188709	rs13107325	*SLC39A8*	C	T	0.928	1.12 (1.08-1.16)	1.60E-08	0.029 (0.013)	0.0249	5.798	6.70E-09
11	121501406	rs10790449	*SORL1*	C	T	0.463	1.06 (1.04-1.08)	1.70E-08	0.002 (0.007)	0.8032	4.532	5.84E-06
16	53809123	rs55872725	*FTO*	C	T	0.599	1.06 (1.04-1.09)	3.60E-09	0.008 (0.007)	0.2491	5.305	1.13E-07
17	43751913	rs1635291	*CRHR1*	A	G	0.754	1.08 (1.05-1.10)	4.50E-10	0.019 (0.008)	0.1258	5.803	6.52E-09

Table S3. eQTL analysis outcomes.

Table provided separately

Table S4. LD between the top eQTL SNP for any eQTL signal and the GWAS SNP.

Lead SNP from UK Biobank and replicated in GERA	eQTL gene symbol	Implicated tissue	Lead SNP for eQTL gene	R2 between SNPs
rs13130794	RFC1	• Brain - Cerebellar Hemisphere<br>• Muscle - Skeletal	rs3736168	0.372
rs13130794	UGDH	• Whole Blood	rs3172642	0.072
rs1260326	AC074117.10	• Brain - Cerebellum<br>• Cells - Transformed fibroblasts<br>• Brain - Cerebellar Hemisphere	rs7602534	0.4626
rs1260326	ATRAID	• Cells - Transformed fibroblasts	rs1978881	0.1391
rs1260326	FNDC4	• Thyroid	rs814295	0.0942
rs1260326	GCKR	• Thyroid	rs6718128	0.0921
rs1260326	KRTCAP3	• Adrenal Gland<br>• Muscle - Skeletal	rs1647285	0.4912
rs1260326	NRBP1	• Skin - Sun Exposed (Lower leg)<br>• Skin - Not Sun Exposed (Suprapubic)<br>• Adipose - Subcutaneous<br>• Small Intestine - Terminal Ileum<br>• Whole Blood<br>• Artery - Tibial<br>• Colon - Transverse<br>• Nerve - Tibial<br>• Cells - Transformed fibroblasts	rs2303370	0.6074
rs1260326	PPM1G	• Muscle - Skeletal	rs11904779	0.4807
rs1260326	SNX17	• Muscle - Skeletal	rs7566052	0.473
rs11214609	TTC12	• Muscle - Skeletal<br>• Esophagus - Gastroesophageal Junction<br>• Esophagus - Muscularis<br>• Skin - Sun Exposed (Lower leg)<br>• Skin - Not Sun Exposed (Suprapubic)<br>• Lung<br>• Heart - Left Ventricle<br>• Colon - Transverse	rs2236709	0.0015

		• Artery - Tibial<br>• Nerve - Tibial<br>• Adipose - Subcutaneous<br>• Heart - Atrial Appendage<br>• Thyroid<br>• Esophagus - Mucosa<br>• Colon - Sigmoid<br>• Adipose - Visceral (Omentum)<br>• Breast - Mammary Tissue<br>• Artery - Aorta<br>• Stomach<br>• Pituitary<br>• Pancreas<br>• Whole Blood<br>• Brain - Frontal Cortex (BA9)<br>• Brain - Cortex<br>• Adrenal Gland		
rs11214609	ANKK1	• Nerve - Tibial<br>• Whole Blood<br>• Lung<br>• Testis<br>• Muscle - Skeletal<br>• Colon - Sigmoid<br>• Adipose - Subcutaneous<br>• Adipose - Visceral (Omentum)<br>• Thyroid<br>• Pancreas<br>• Heart - Atrial Appendage<br>• Heart - Left Ventricle<br>• Colon - Transverse	rs10891544	0.4733
rs11214609	RP11-159N11.4	• Skin - Not Sun Exposed (Suprapubic)<br>• Esophagus - Muscularis<br>• Lung<br>• Nerve - Tibial	rs3897583	0.0035

Table S5. Variant-trait significant outcomes from PheWAS.

SNP	SNP locus	Trait	P-value
rs1229984	*ADH1B*	F10 Mental and behavioural disorders due to use of alcohol	1.29×10^{-19}
		E78 Disorders of lipoprotein metabolism and other lipidaemias	1.44×10^{-7}
		I10-I15 Hypertensive diseases	1.44×10^{-7}
		I10 Essential (primary) hypertension	1.89×10^{-7}
		F10-F19 Mental and behavioural disorders due to psychoactive substance use	3.40×10^{-7}
		M10 Gout	8.83×10^{-7}
		E70-E90 Metabolic disorders	3.08×10^{-6}
		K70 Alcoholic liver disease	1.12×10^{-5}
rs1260326	*GCKR*	E78 Disorders of lipoprotein metabolism and other lipidaemias	7.63×10^{-17}
		M10 Gout	3.72×10^{-14}
		E70-E90 Metabolic disorders	3.59×10^{-12}
		K80-K87 Disorders of gallbladder, biliary tract and pancreas	9.08×10^{-12}
		K80 Cholelithiasis	1.25×10^{-11}
		E10-E14 Diabetes mellitus	1.42×10^{-10}
		E11 Non-insulin-dependent diabetes mellitus	2.51×10^{-10}
		I20-I25 Ischaemic heart diseases	1.39×10^{-5}
rs13107325	*SLC39A8*	M15-M19 Arthrosis	9.03×10^{-8}
		M75 Shoulder lesions	5.04×10^{-7}
		M13 Other arthritis	8.83×10^{-7}
		M20-M25 Other joint disorders	3.14×10^{-6}
		I10 Essential (primary) hypertension	4.01×10^{-6}
		I10-I15 Hypertensive diseases	4.13×10^{-6}
		K44 Diaphragmatic hernia	1.08×10^{-5}

Table S6. Variants at 5 × 10^{-6} and submitted to the Reactome Knowledgebase.

locus	SNP	P
ADH1B	rs1229984	3.30E-36
KLB	rs13130794	2.60E-10
CRHR1	rs1635291	4.50E-10
FTO	rs55872725	3.60E-09
SLC39A8	rs13107325	1.60E-08
SORL1	rs10790449	1.70E-08
BANK1	rs76062146	1.90E-08
GCKR	rs1260326	2.60E-08
FBXO11	rs4952896	6.80E-08
RP11-89K21.1	rs1004787	2.00E-07
DRD2	rs11214609	2.10E-07
DNAH7	rs62203362	8.40E-07
DCC	rs113986473	9.20E-07
TCF4	rs117316028	9.50E-07
SUGCT	rs78619948	1.10E-06
MED22	rs621907	1.10E-06
PTPRE	rs12217409	1.20E-06
DNAH5	rs6874349	1.30E-06
RUNX1T1	rs17747233	1.30E-06
NOX4	rs12285366	1.60E-06
BANK1	rs201081507	1.70E-06
AC006159.3	rs12673233	1.70E-06
FAIM2	rs7132908	1.90E-06
LRRTM4	rs35525922	1.90E-06
DGKQ	rs61757581	2.00E-06
CPQ	rs1075479	2.10E-06
DENND1A	rs4838060	2.20E-06
NRXN3	rs78668413	3.20E-06
TLK2	rs143310582	3.40E-06
ESR1	rs35882398	3.40E-06

CCDC88A	rs141392636	3.50E-06
FMNL2	rs11692786	3.60E-06
PCTP	rs187671201	3.80E-06
ELTD1	rs188522508	3.90E-06
LINC00877	rs139430561	4.00E-06
MLF2	rs2286729	4.50E-06
GRM3	rs192141105	4.60E-06

Table S7. Mendelian randomization results for nominally significant outcomes in the IVW analysis and FDR outcomes using the IVW method.

Outcome	*B*	se	P-value (IVW)	FDR P-value
Ischaemic stroke	-63.87	18.43	0.0005	0.0485
Insulin sensitivity index	-3.53	1.37	0.0098	0.3126
AUC insulin	2.91	1.19	0.0145	0.3126
Lung adenocarcinoma	1.81	0.76	0.0168	0.3126
Incremental insulin at 30 minutes	3.03	1.31	0.0212	0.3126
Nucleus accumbens volume	-91.59	40.00	0.0220	0.3126
Extreme waist-to-hip ratio	-5.18	2.31	0.0252	0.3126
Putamen volume	-449.85	203.65	0.0272	0.3126
Eczema	-1.19	0.59	0.0429	0.3593
Lung cancer	1.04	0.52	0.0450	0.3593
Transferrin	-1.52	0.76	0.0468	0.3593
Insulin at 30 minutes	2.28	1.16	0.0495	0.3593

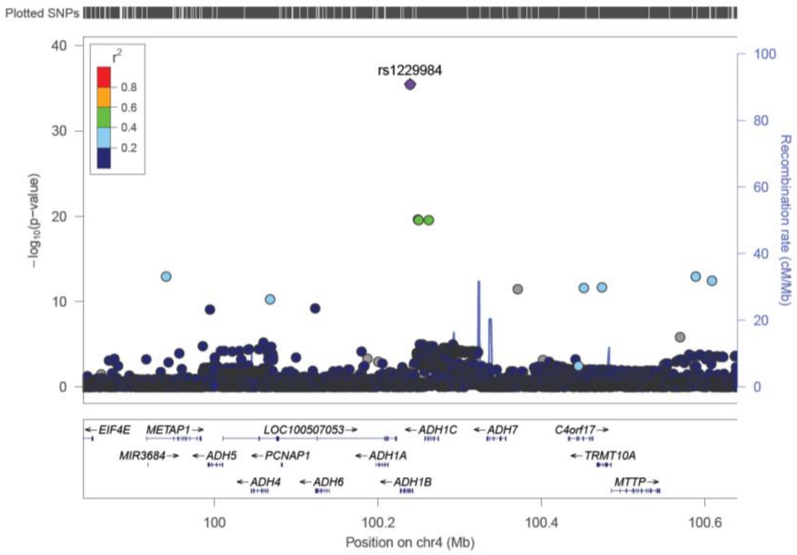

Figure S1a: LocusZoom plot for rs1229984

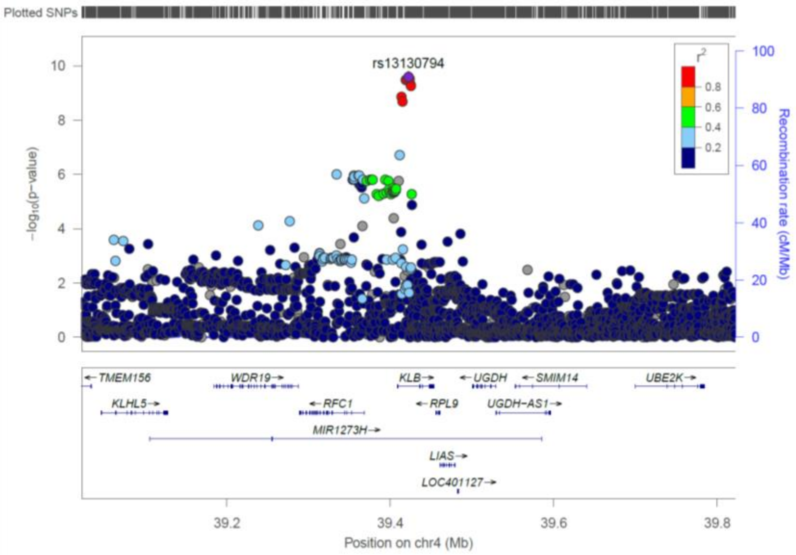

Figure S1b: LocusZoom plot for rs13130794

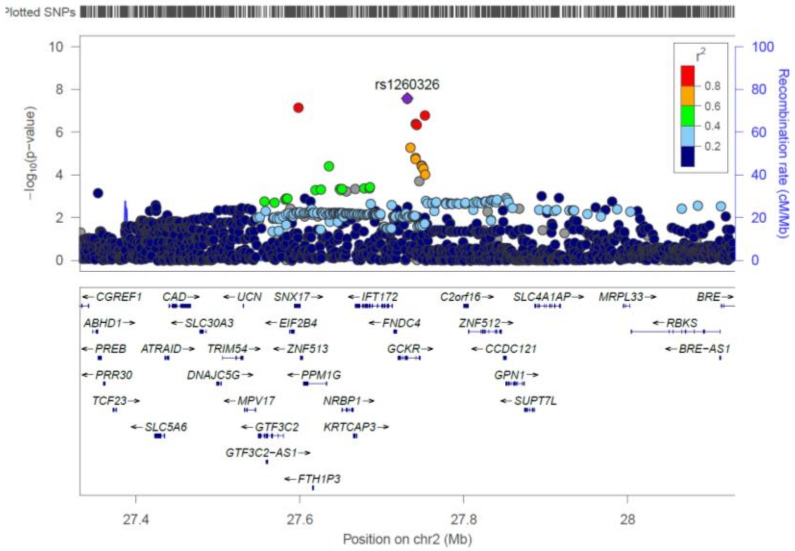

Figure S1c: LocusZoom plot for rs1260326

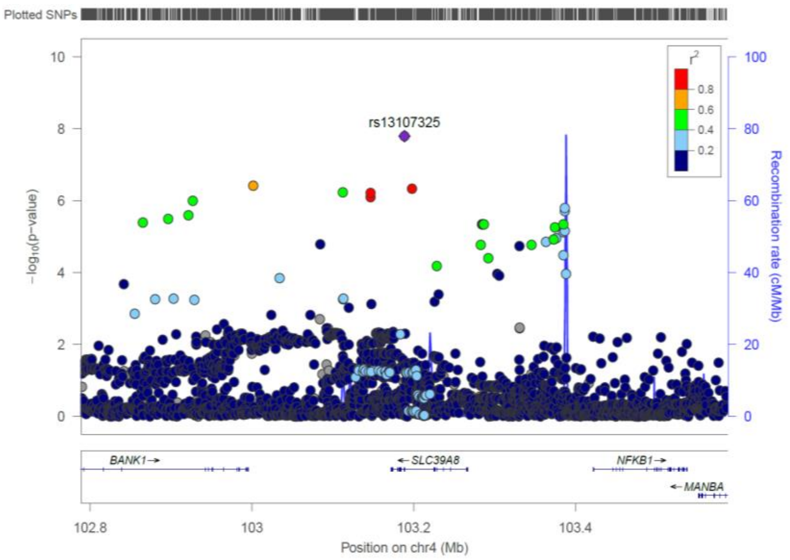

Figure S1d: LocusZoom plot for rs13107325

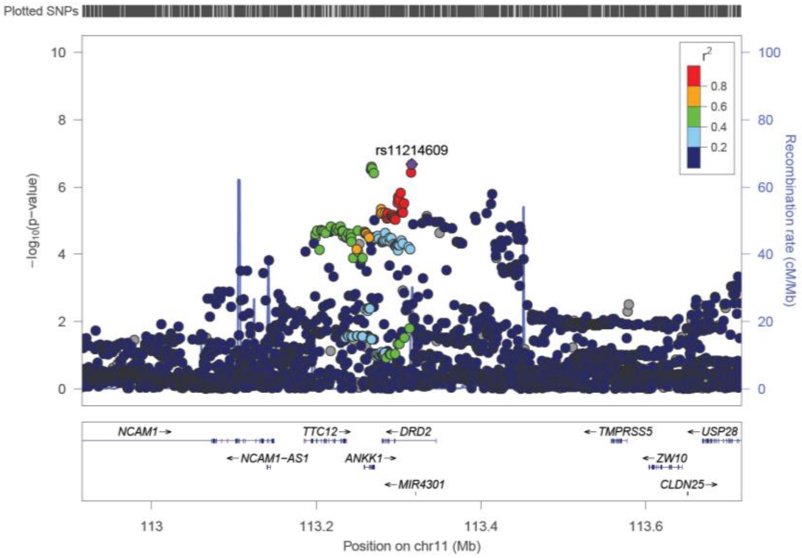

Figure S1e: LocusZoom plot for rs11214609

Fig. S1. LocusZoom plots for lead SNPs from GWAS on alcohol phenotype in the entire cohort.

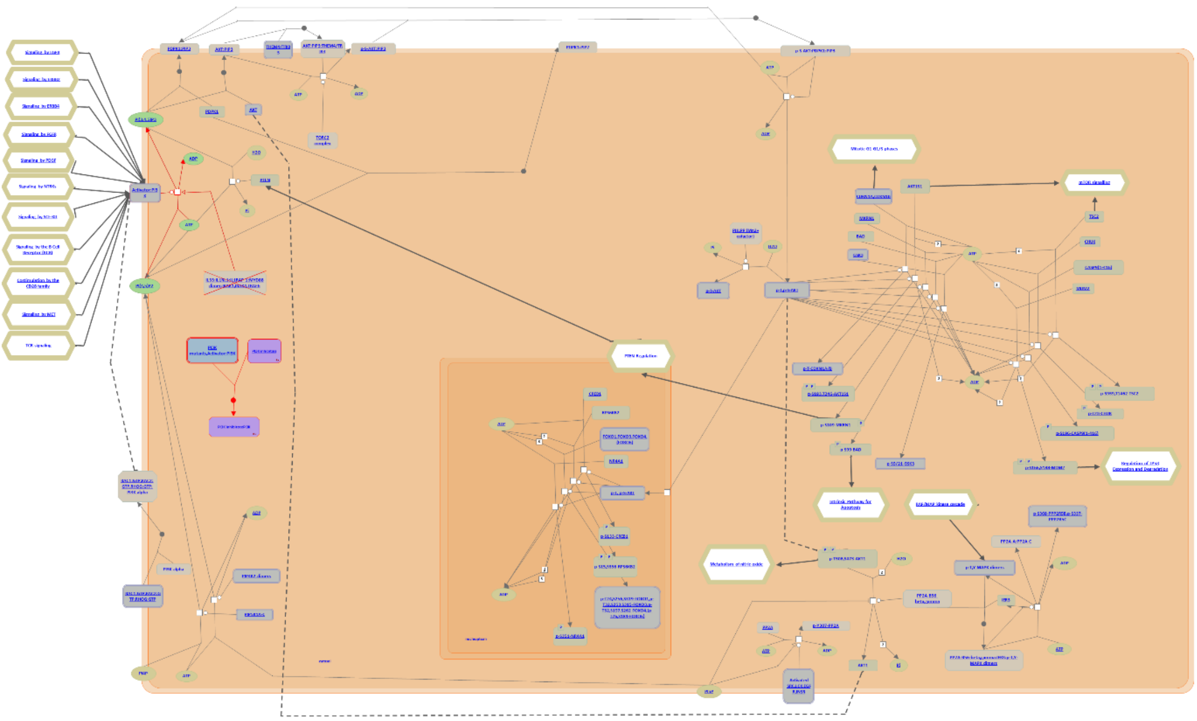

Fig. S2. Constitutive signaling by aberrant PI3K in cancer.

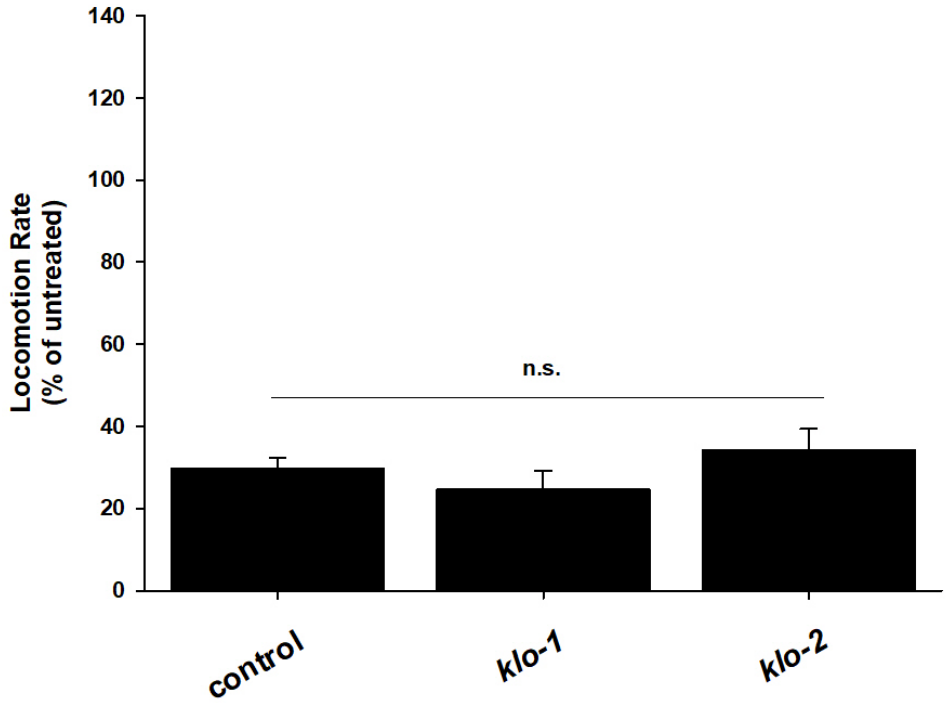

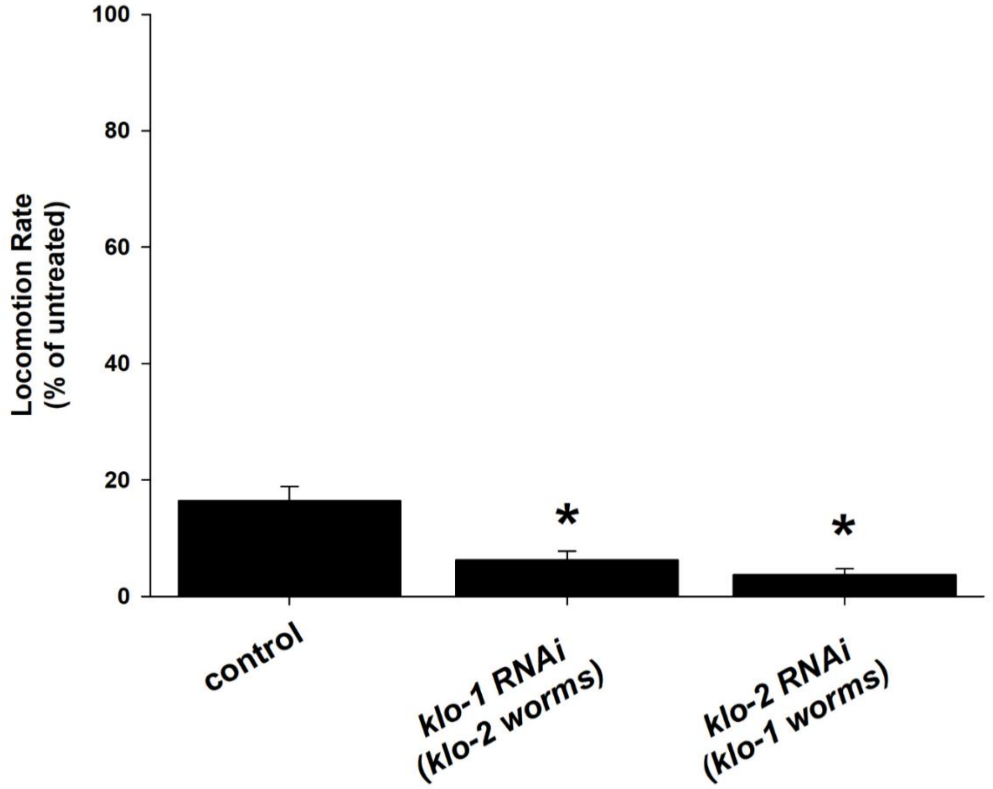

Fig. S3. Individual *C. elegans* β-Klotho genes outcomes. Individual *C. elegans* β-Klotho genes outcomes: **A**) Individual *C. elegans* Beta klotho genes do not alter alcohol phenotypes. In comparison to controls (Bristol N2), *loss-of-function* mutations in *klo-1* or *klo-2* genes do not affect the locomotion rate of worms treated with ethanol. Results are presented normalised to locomotion of untreated worms (basal locomotion rate: 99.03±1.47 (Bristol N2 controls), 98.4±2.89 (*klo-1*), 113.2±2.12 (*klo-2*)); **B**) RNAi of individual *C. elegans* Beta klotho genes. RNAi knockdown of worm beta klotho genes in the *loss-of-function* mutant strain of the other worm beta klotho gene. Results are presented as locomotion of worms treated with ethanol normalised to untreated worms (basal locomotion rate: 87.63±21.6 (empty vector control), 111.10±3.15 (*klo-1* RNAi in *klo-2* worms), 117.03±3.30 (*klo-2* RNAi in *klo-1* worms)).
